# Patient, tumour, and dosimetric factors influencing survival in non-small cell lung cancer patients treated with stereotactic ablative body radiotherapy

**DOI:** 10.1093/bjro/tzae028

**Published:** 2024-09-25

**Authors:** Minal Padden-Modi, Yevhen Spivak, Ian Gleeson, Andrew Robinson, Kamalram Thippu Jayaprakash

**Affiliations:** Department of Oncology, Addenbrooke’s Hospital, Cambridge University Hospitals NHS Foundation Trust, Cambridge, CB2 0QQ, United Kingdom; Department of Oncology, Addenbrooke’s Hospital, Cambridge University Hospitals NHS Foundation Trust, Cambridge, CB2 0QQ, United Kingdom; Department of Medical Physics, Addenbrooke’s Hospital, Cambridge University Hospitals NHS Foundation Trust, Cambridge, CB2 0QQ, United Kingdom; Department of Medical Physics, Addenbrooke’s Hospital, Cambridge University Hospitals NHS Foundation Trust, Cambridge, CB2 0QQ, United Kingdom; Department of Oncology, Addenbrooke’s Hospital, Cambridge University Hospitals NHS Foundation Trust, Cambridge, CB2 0QQ, United Kingdom

**Keywords:** non-small cell lung cancer, stereotactic ablative body radiotherapy, survival

## Abstract

**Objectives:**

We aimed to analyse clinical outcomes of peripheral, early-stage non-small cell lung cancer (NSCLC) patients treated with stereotactic ablative body radiotherapy (SABR), and evaluate potential patient, tumour, and dosimetric variables influencing survival.

**Methods:**

Data were collected retrospectively from patients treated between September 2012 and December 2016 and followed up until January 2021. Patient demographics, tumour characteristics, SABR dosimetric parameters, and survival data were collected from electronic patient medical records. Descriptive statistics were performed, and SPSS software was used for survival analysis.

**Results:**

Eighty-nine patients were included of whom 49.5% were male and 50.5% female. Median age was 74 years. 98.8% of patients had T1-2 tumours and 89.9% underwent 55 Gy in 5 fractions. Median overall survival time was 58.7 months. On uni- and multi-variate analysis, neither patient nor tumour variables showed association with overall survival. However, planning target volume (PTV) and minimum dose to PTV correlated with overall survival. There was a signal for association between mean lung dose and overall survival on multivariate analysis.

**Conclusions:**

Our long-term results show SABR is an effective treatment for peripheral, early-stage NSCLC with excellent overall survival, comparable to other series. Our study found only the PTV and minimum dose to PTV had an impact on overall survival, which demonstrates the importance of generating optimal SABR plans.

**Advances in knowledge:**

Our work identified lung SABR dosimetric parameters that correlate with survival, which illustrates the importance of producing optimal lung SABR plans.

## Introduction

Lung cancer continues to be the biggest cause of cancer-related death, accounting for in excess of 350 000 deaths per year in Europe despite advances in treatments. Around a fifth of all cancer deaths are because of lung cancer and it is most prevalent in the elderly population.^1^^,^^2^

Treatment options for patients with peripheral, early-stage non-small cell lung cancer (NSCLC) include surgery and radical radiotherapy such as stereotactic ablative body radiotherapy (SABR), depending on their operability and the patient’s wishes. Surgery is the gold standard treatment for operable early-stage NSCLC; however, there is now much data supporting the use of SABR to be comparably effective in both overall survival (OS) and progression free survival (PFS).^3^^,^^4^ It is therefore well established, that for early-stage NSCLC, SABR is the standard treatment to those patients who are not fit enough for resection or refuse surgery.^5^

SABR offers an ablative dose of radiation to a finite, peripheral or centrally located target, to minimize dose to organs at risk, which, if not controlled, could lead to treatment related mortality. SABR is preferred in patients who have co-morbidities, poor performance status or inoperable tumours, where it is an alternative, which is convenient for patients, non-invasive and highly effective.^4^

The effectiveness of SABR appears to depend on several variables. A meta-analysis performed by Zhang et al.^6^ suggests that the biologically effective dose (BED) delivered to an early-stage NSCLC may affect OS. SABR offers highly effective treatment to those with multiple comorbidities; however, extra caution needs to be exercised when treating patients with interstitial lung fibrosis^7^ and ultra-central tumours.^8^ SABR fractionations used in clinical practice are largely based on the tumour location with respect to organs at risk, defined as risk zones and various prescription and planning practices exist. There are clinical and dosimetric variables that may determine survival of these patients treated with SABR. Our study aims to investigate whether patient, tumour, dosimetric variables impact on longer-term outcomes in peripheral, early-stage NSCLC patients treated with SABR.

## Methods

All consecutive patients with peripheral, early-stage NSCLC treated with SABR between September 2012 and December 2016 at our institution with a minimum follow-up of 4 years were included in this study. Patients were selected for SABR based on the following criteria: primary lung cancer (T1-2a N0 and occasional T3 N0); ECOG (Eastern Cooperative Oncology Group) performance status 0–2 and occasionally 3; patient not suitable for or who decline surgery; lesion outside the “no-fly zone”; local Lung MDT (multidisciplinary team) agreement that lesion represents a primary lung cancer and is suitable for SABR (confirmed NSCLC on histology AND/OR positive PET scan AND/OR growth on serial CT scans) and absence of significant interstitial lung disease.

**Figure 1.**
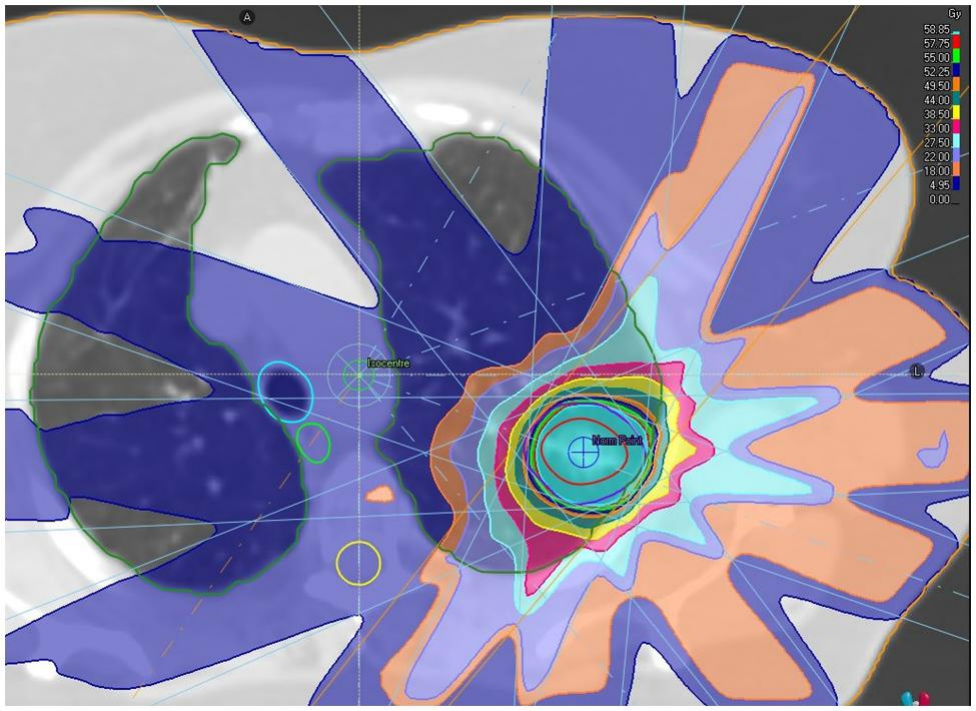
Example SABR plan shown in radiation dose gradient. Organs at Risk (OAR) shown are lungs, spinal cord, oesophagus, and proximal airways. The dose prescription used was 55 Gy/5#.

Patients’ electronic medical records from EPIC (Epic Systems Corporation, Verona, Wisconsin, USA) and MOSAIQ Oncology Information System (Elekta, Stockholm, Sweden) were used to collect patient demographics, tumour characteristics, treatment details, and patient outcomes. 3D conformal planning and volumetric modulated radiotherapy (VMAT) techniques was used to deliver SABR, with planning objectives as per the UK SABR consortium guidelines^9^ available at that time (Figure 1). On-going local surveillance was carried out every 3 to 6 months initially in appropriate patients and then annually until year 5 of follow-up by local oncology teams. Recurrence was detected on cross sectional imaging studies, defined in Table 1.

**Table 1.** Definition of recurrence.

Recurrence type	Definition
Local	Progression of lesion within treatment field
Regional	Regional lymph nodes
Distant	Beyond regional progression

This project was registered with and approved as a quality improvement project by our institutional clinical audit department (project identification number: PRN9422).

Descriptive statistics were performed, and SPSS software was used for analysing clinical outcomes. The key clinical outcome that was analysed was OS. OS probabilities were estimated using Kaplan–Meier’s survival analysis. Univariate and multivariate Cox regression models were employed to study the impact of patient, tumour, and dosimetric factors on OS.

## Results

### Patient characteristics, tumour factors, and dosimetric parameters studied

Patient characteristics, tumour factors, and dosimetric parameters of 89 patients included in this study are summarized in Table 2. The median age of patients was 74 years (ranging between 50 to 90). Histological confirmation rate was 51.7% (*n* = 46), of which, 69.55% had adenocarcinoma (*n* = 32), 28.26% had squamous cell carcinoma (SCC) (*n* = 13), and 2.1% had small cell lung carcinoma (*n* = 1). 60.7% had T1a disease (*n* = 55), 22.5% had T1b (*n* = 20), and 13.49% had T2a stage disease (*n* = 12).

**Table 2.** Patient characteristics, tumour factors and dosimetric parameters.

Patient characteristics and tumour factors	Category	*n*	%
Total patient number		89	
Age (years)	Median	74.24	
	Range	50–90	
Gender	Male	44	49.43
	Female	45	50.57
Smoking status	Current	13	14.60
	Ex	61	68.54
	Never	8	8.99
	Missing	7	7.87
Performance status	0	8	8.99
	1	40	44.94
	2	32	35.96
	3	4	4.49
	Missing	5	5.62
T Stage	T1a	55	60.68
	T1b	20	22.47
	T1c	0	0
	T2a	12	13.49
	Missing	2	2.24
Histological diagnosis	Yes	46	51.69
	No	42	47.19
	Missing	1	1.12
Dose and fractionation	55 Gy/5#	80	89.89
	60 Gy/8#	8	8.99
	Other	1	1.12
Tumour location	RUL	35	39.32
	RML	1	1.12
	RLL	2	2.24
	LUL	29	32.58
	LLL	14	15.73
Laterality	Right	46	51.68
	Left	43	49.32
Charlton comorbidity index	Median	6	
	Range	4-14	
CT appearance	Solid	71	79.77
	Ground glass	9	10.12
	Cavitating	4	4.49
	Missing	5	5.62

Dosimetric parameters	Median	Range	

PTV volume (mL)	21.48	7.59–85.05	
55 Gy/5#	48.1	7.59–85.05	
60 Gy/8#	23.08	15–41.06	
R100%	1.14	0.21–1.63	
R50%	6	4.1–13.9	
Maximum dose to >2 cm from PTV (Gy)	31.44	21.1–45.18	
Mean lung dose (Gy)	3.3	1.68–6.3	
Lungs V20Gy (%)	4.15	1.29–8.7	
PTV maximum dose Gy (%)	74.85	6319.919–83.8	
PTV minimum dose Gy (%)	47.8	329.566–71.19	
PTV V90% dose (%)	99.9	30.27–100	
PTV V100% dose (%)	95.0215	20.8–99.66	

GTV = gross tumour volume; PTV = planning target volume; R100 = volume receiving 100% of the dose/PTV volume; R50 = volume receiving 50% of the dose/PTV volume; V20 = % volume of total lung minus GTV receiving 20 Gy; PTV V100% and PTV V90% = percent of the PTV receiving 100% and 90% of the prescription dose.

### Overall survival

**Figure 2.**
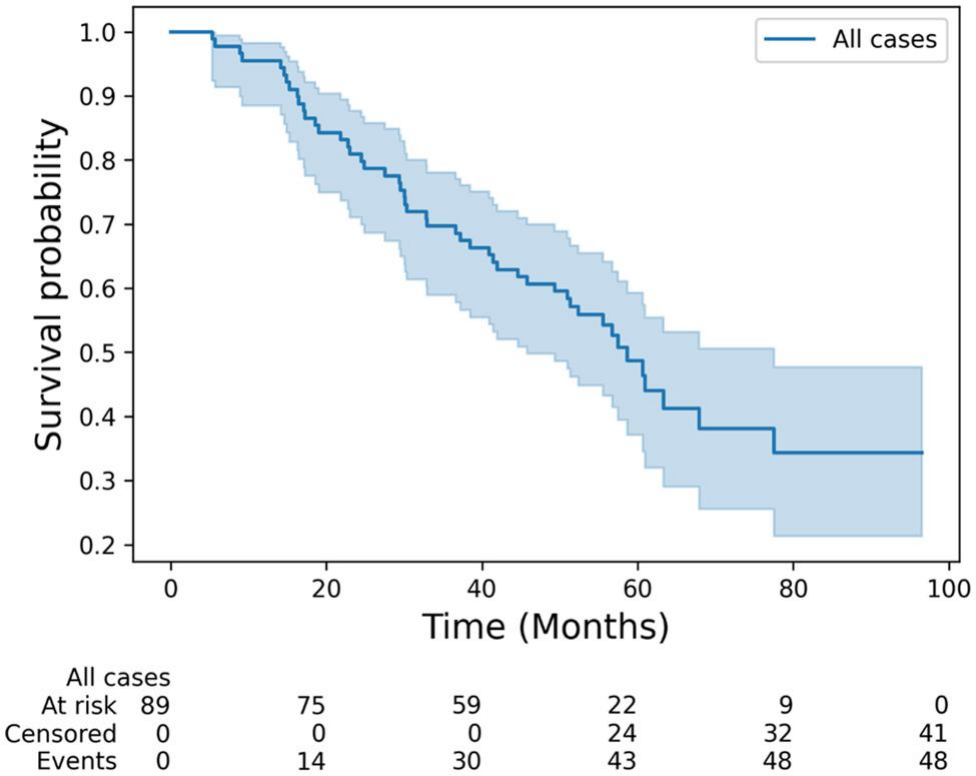
Overall survival, Kaplan–Meier survival analysis.

At the time of analysis, 46% (*n* = 41) of patients were still alive. Patients had a median overall survival of 58.7 months (Figure 2).

### Variables influencing survival

#### Patient characteristics

14.60% were current smokers, 68.54% were ex-smokers, and 8.99% were never smokers. In 7.87% of cases, there was no smoking history available. Both univariate and multivariate Cox proportional hazard analysis confirmed no statistically significant differences in hazard ratio (HR) between smokers and non-smokers. Patients with a performance status (PS) of 0, 1, 2, and 3 were distributed 8.99%, 44.94%, 35.96%, and 4.49%, respectively. There was a signal towards association between PS and overall survival with univariate analysis (HR 1.424 [95% CI 0.99–2.04]; *P* = 0.057); however, with multivariate analysis, considering other potential confounding factors, this association was lower (HR of 1.307 [95% CI 0.76–2.24]; *P* = 0.33). The median Charlton comorbidity index (CCI) was 6 and there was no statistically significant relationship found between CCI and OS (Tables 3 and 4).

**Table 3.** Univariate Cox proportional hazard analysis correlating variables with overall survival.

Variables		Overall survival
	HR (95% CI)	*P*-value
Age	1.014 (0.978–1.052)	0.448
Gender	0.797 (0.451–1.411)	0.437
Smoking status	0.929 (0.542–1.592)	0.789
Performance status	1.424 (0.99–2.04)	0.057
Histology	1.129 (0.754–1.689)	0.556
Dose 8# or 5#	0.948 (0.891–1.01)	0.099
Lobe	1.093 (0.911–1.31)	0.339
Laterality	1.606 (0.907–2.8)	0.104
Charlton comorbidity index	1.029 (0.881–1.2)	0.715
CT appearance	0.999 (0.997–1.001)	0.164
Biopsy	0.761 (0.419–1.379)	0.368
PTV volume (mL)	1.020 (1.004–1.035)	0.012
R100	2.456 (0.439–13.727)	0.306
R50	0.877 (0.731–1.053)	0.160
Maximum dose to >2 cm from PTV (Gy)	1.022 (0.965–1.082)	0.464
Mean lung dose (Gy)	0.897 (0.668–1.205)	0.470
Lung V20 (%)	1.183 (0.841–1.183)	0.978
Maximum dose to PTV	0.992 (0.96–1.025)	0.640
Minimum dose to PTV	0.941 (0.893–0.991)	0.022
PTV V90%	1.022 (0.967–1.081)	0.443
PTV V100%	1.012 (0.981–1.044)	0.451

**Table 4.** Multivariate Cox proportional hazard analysis correlating variables with overall survival.

Variables		Overall survival
	HR (95% CI)	*P*-value
Age	0.995(0.93–1.06)	0.888
Gender	1.969(0.61–6.32)	0.255
Smoking status	0.853(0.38–1.91)	0.70
Performance status	1.307(0.76–2.24)	0.330
Lobe	0.635(0.28–1.4)	0.261
Laterality	3.870(0.24–61.7)	0.338
Charlton comorbidity index	1.058(0.81–1.38)	0.680
CT appearance	1.0(0.99–1.00)	0.837
Biopsy	1.259(0.41–3.84)	0.686
PTV volume (mL)	1.069(1.02–1.1)	0.001
R100	0.132(0.0–82.2)	0.538
R50	1.244(0.86–1.79)	0.246
Maximum dose to >2 cm from PTV (Gy)	0.948(0.84–1.06)	0.362
Mean lung dose (Gy)	0.390(0.15–1.01)	0.053
Lung V20 (%)	1.103(0.72–1.68)	0.648
Maximum dose to PTV	1.011(0.97–1.05)	0.595
Minimum dose to PTV	0.785(0.66–0.92)	0.004
PTV V90%	1.041(0.92–1.18)	0.533
PTV V100%	1.065(0.93–1.2)	0.354

#### Tumour factors

Univariate analysis showed that the HR for OS in patients with a histological diagnosis was 0.76 (95% CI 0.419–1.379; *P* = 0.368), compared to patients who were diagnosed with imaging, showing histological confirmation had no statistically significance impact in improving survival. However, in those patients with a radiological diagnosis, 48% had died which is in contrast to patients with adenocarcinoma of whom 53% had died and in those with SCC 73% had died. Moreover, when analysing recurrences, following SABR adenocarcinoma recurred in 34.7% (16 of 46 patients) and SCC recurred in 40% (6 of 15 patients).

#### Dosimetric parameters

The median planning target volume (PTV) was 21.56 cm^2^ (range, 7.59–85.05). The median PTV was 48.1 cm^2^ (7.59–85.05) for 55 Gy in 5 fractions and 23.08 cm^2^ (15–41.06) for 60 Gy in 8 fractions. On univariate and multivariate analysis, the HR for OS was 1.02 (*P* = 0.012) and 1.069 (*P* = 0.001), respectively, suggesting that a larger PTV was associated with poorer survival. Univariate and multivariate analysis suggested no significant relationship between R100%, R50%, and D2cm (maximum dose to >2 cm from PTV) and overall survival.

Maximum dose to PTV (Gy) did not show a statistically significant HR for OS through either univariate or multivariate analysis. There was a very strong association between minimum dose to PTV and overall survival (HR 0.941; *P* = 0.022 [univariate analysis], and HR 0.78; *P* = 0.004 [multivariate analysis]).

The median PTV V90% (PTV receiving 90% of the prescribed dose) was 99.9% (range 30.27–100%). Univariate analysis did not show a statistically significant difference between survival and PTV V90% (HR 1.022 [95% CI 0.967–1.081]; *P* = 0.443). Similarly, no statistical significance was demonstrated with multivariable analysis (HR 1.0065 [95% CI 0.93–1.2]; *P* = 0.354).

The proportion of the lung volume receiving >20 Gy (V20 in %) was 4.15 (1.29–8.7) and this did not significantly affect OS through univariate and multivariate analysis. Median mean lung dose (MLD) (Gy) was 3.3 (1.69–6.3). Univariate analysis did not to show a statistically significant association between survival and MLD (HR 0.897 [95% CI 0.668–1.2050]; *P* = 0.47). However, on multivariate analysis the HR was 0.39 (95% CI 0.15–1.01; *P* = 0.053) suggesting a stronger relationship between MLD and mortality.

#### Treatment tolerance, toxicity, and recurrence rates

SABR treatment was well tolerated, with only one patient not completing treatment due to an unrelated reason. No recurrence was seen in 67% of patients. In those who did have recurrence, 9% of patients developed local recurrence, 5.6% had regional recurrence, 14.6% had distant recurrent disease. Late toxicities were seen in 19 patients (21.3%).

## Discussion

Data from our retrospective study favourably compared to the outcomes of patients with early-stage NSCLC treated with SABR in other studies, confirming SABR as an effective treatment modality in this group of patents.^10^^,^^11^ We studied key patient, tumour, and dosimetric variables that may have an influence on survival.

Our study showed that PS might be a good predictor for poor outcomes; however, this is not statistically significant in our data after multivariate analysis, and may be attributable to several other associated variables, which deemed the association difficult to quantify. This association is plausible, as poor PS patients do not do well. Further analysis with a larger sample size into PS and OS is needed to further characterize this potential effect. Our study indicates that the use of SABR in those patients with SCC needs further survival analysis through larger population studies, as our data is suggestive of poorer outcomes in SCC. This could partly be explained by the morphological appearance of SCC where some may present with a cavitating lesion that could represent hypoxic tumour and this morphology could in turn have an impact on the dosimetry as well as reduce sensitivity to radiotherapy.^12^

Both univariate and multivariate analysis suggested that a larger PTV was associated with a poorer outcome. This may correlate with increased tumour burden and staging. However, tumour staging independently did not appear to have a significant association with poorer outcomes as Schonewolf et al.^13^ reported no relationship between tumour size and outcomes; therefore, association between PTV volume and overall survival warrants further investigation. Similar to Saha et al.’s^10^ study analysing OS in patients treated with SABR, we also noted that reduced number of fractions tended to be used to treat patients with a larger PTV volume. However, in contrast to their results, we did not observe correlation between R100 and R50, indicating these dosimetric parameters need further investigation.

Our findings showed that patients receiving a higher minimum dose to the PTV was associated with increased overall survival and higher dose delivery to 90% of the PTV (PTV V90%) resulted in a better outcome. These findings demonstrate the importance of producing optimal coverage in SABR plans to deliver planned dose to improve patients’ survival. From a dosimetry point of view, optimal target coverage may not always be possible due to very low tissue density, which can lead to lack of dose build up which is a common issue that may limit our ability to achieve our optimal lung SABR planning objectives.

We noted that patients receiving a higher MLD had an increase in median OS, which contrasts with what we would expect. However, this finding needs to be interpreted with caution as this is a small patient cohort study and there could be other unmeasured, confounding factors such as severe pre-existing lung diseases such as COPD (chronic obstructive pulmonary disease) that could explain this finding. Therefore, a larger study would help to investigate this relationship further.

When reviewing toxicities, 19 patients out of 89 (21%) developed late toxicities. Although we do not have grading of toxicities, the proportion of toxicities is notably higher compared to another similar study by Wood et al.^14^ This highlights the important of robust follow-up data collection where patient reported outcomes could play a vital role.

Our study represents a patient population treated with a homogenous SABR fractionation and representing real world outcomes of SABR in early-stage NSCLC. Our long-term follow-up of patients can also be added to the list of strengths, as it allowed us to better understand longer-term outcomes in the patients reviewed. Our study has limitations of a retrospective study of a small patient cohort and there may be unmeasured confounders that have led to the results we have observed. Our study has limited data on recurrence and toxicities, which are very important for lung SABR studies. Nevertheless, our data further helps to improve our understanding of potential patient, clinical, and dosimetric variables that may influence outcomes for these patients.

## Conclusion

Survival of patients in our cohort are comparable to that of larger trials and population studies.^10^^,^^15^^,^^16^ We identified several dosimetric parameters that may have association with survival of patients with peripheral, early-stage NSCLC treated with SABR, highlighting the importance of generating optimal lung SABR plans to improve outcomes for these patients.
